# Negative Priming Effect on Organic Matter Mineralisation in NE Atlantic Slope Sediments

**DOI:** 10.1371/journal.pone.0067722

**Published:** 2013-06-28

**Authors:** Evangelia Gontikaki, Barry Thornton, Veerle A. I. Huvenne, Ursula Witte

**Affiliations:** 1 Institute of Biological and Environmental Sciences, Oceanlab, University of Aberdeen, Aberdeen, United Kingdom; 2 The James Hutton Institute, Aberdeen, United Kingdom; 3 National Oceanography Centre, Southampton, United Kingdom; Dowling College, United States of America

## Abstract

The priming effect (PE) is a complex phenomenon which describes a modification (acceleration or retardation) in the mineralisation rate of refractory organic matter (OM) following inputs of labile material. PEs are well-studied in terrestrial ecosystems owing to their potential importance in the evolution of soil carbon stocks but have been largely ignored in aquatic systems despite the fact that the prerequisite for their occurrence, i.e. the co-existence of labile and refractory OM, is also true for sediments. We conducted stable isotope tracer experiments in continental margin sediments from the NE Atlantic (550–950 m) to study PE occurrence and intensity in relation to labile OM input. Sediment slurries were treated with increasing quantities of the ^13^C-labelled diatom *Thalassiosira rotula* and PE was quantified after 7, 14 and 21 days. There was a stepwise effect of diatom quantity on its mineralisation although mineralisation efficiency dropped with increasing substrate amounts. The addition of diatomaceous OM yielded a negative PE (i.e. retardation of existing sediment OM mineralisation) at the end of the experiment regardless of diatom quantity. Negative PE is often the result of preferential utilisation of the newly deposited labile material by the microbial community (“preferential substrate utilization”, PSU) which is usually observed at excessive substrate additions. The fact that PSU and the associated negative PE occurred even at low substrate levels in this study could be attributed to limited amounts of OM subject to priming in our study area (∼0.2% organic carbon [OC]) which seems to be an exception among continental slopes (typically >0.5%OC). We postulate that PEs will normally be positive in continental slope sediments and that their intensity will be a direct function of sediment OC content. More experiments with varying supply of substrate targeting C-poor vs. C-rich sediments are needed to confirm these hypotheses.

## Introduction

Continental margin sediments (200–2000 m) cover merely 9% of the ocean’s seafloor but are responsible for ∼30% of the global benthic mineralisation [1]. Understanding when these systems act as a source vs. sink of carbon (C) is thus of primary importance if we are to produce reliable global C budgets and predict the effects of future perturbations on the global C cycle. The chemical nature of organic matter (OM) is thought to be one of the major controls on the degradation/preservation balance in sediments [2], [3]. Phytoplanktonic and macroalgal detritus are sources of highly reactive OM in surface sediments [4], [5], [6], whereas inputs of land-derived OM, microbial reworking of labile OM into less reactive forms and the accumulation of bacterial cell wall components contribute to the more refractory OM pool in marine sediments [7], [8], [9].

Labile and refractory OM pools degrade at different rates but not independently [10]. However, the interactions between these pools and their effect on C cycling are still poorly understood and most often ignored in global change models [11]. The term “priming effect” (PE) has been used to describe such links between labile and refractory OM pools in soils and refers to a modification, usually an increase, in the degradation rate of soil OM after input of a labile substrate. The mechanisms involved in PEs are not completely understood but theoretical approaches suggest that competitive or mutualistic interactions between labile and refractory OM degrading microbes may control the occurrence and intensity of PEs [12], [13]. The potential importance of PEs in CO_2_ evolution in response to anthropogenic perturbations and climate change has attracted considerable attention to these phenomena among soil scientists [14], [15]. Based on studies in terrestrial ecosystems, we know that PEs can be positive (labile OM addition increases soil OM mineralisation) or negative (labile OM addition decreases soil OM mineralisation) depending on the quantity and quality of the added substrate, nutrient levels, soil type and microbial community structure [14], [15], [16], [17]. Our knowledge on PEs in aquatic systems is limited; Hee et al. [18] provided evidence for stimulated hydrolysis of relic (unreactive) sedimentary OM at the presence of fresh OM under anoxic conditions; In addition, the interaction between labile and refractory OM pools has been shown to enhance nitrogen cycling in marine sediments [19], and the growth and respiration of bacterioplankton [20]. In the first experimental study of PEs using stable isotope labelled substrates, van Nugteren et al. [21] reported an increase in the mineralisation of existing sedimentary OM (positive PE) after deposition of phytoplankton-derived material in intertidal and subtidal estuarine sediments. Despite the so far limited number of PE studies in aquatic systems, awareness of the significance of these phenomena for C and nutrient cycling is increasing [11], [22] and an upsurge of interest for PEs among aquatic scientists seems to be imminent.

Sediment metabolism in deep water sediments is mainly supported by phytoplankton detritus sinking from surface waters. Benthic C cycling studies using artificial pulses of isotopically-labelled marine algae, have repeatedly demonstrated the “awakening” of benthic communities at the arrival of this labile material as an overall increase in benthic metabolism [23], [24], [25], [26]. However, whether this increase in sediment metabolism was coupled with priming phenomena is unknown. This is the first study to specifically address the issue of priming in continental slope sediments and was inspired by Guenet et al. [11] who hypothesized that under oligotrophic conditions, such as those prevailing in the deep sea, the occurrence of labile algal-derived OM would stimulate microbial production of extracellular enzymes and maximise the refractory OM mineralisation to reduce nutrient limitation (positive PE). We conducted stable isotope tracer experiments (ITEs) in continental slope sediments of the NE Atlantic (500–1000 m) using increasing quantities of ^13^C-labelled diatom detritus to test the hypotheses that: a) the addition of highly reactive diatom OM will enhance the mineralisation of existing sedimentary OM (positive PE), b) increasing amounts of diatom OM will result in stronger PE intensity [14], [27] and c) PE intensity will become more pronounced with increasingly oligotrophic conditions from shallow- to deep-water stations. PE quantification was based on measurements of total CO_2_ and ^13^CO_2_ evolution in experimental vs. control slurry incubations.

## Materials and Methods

### Study Site and Sediment Sampling

**Figure 1:**
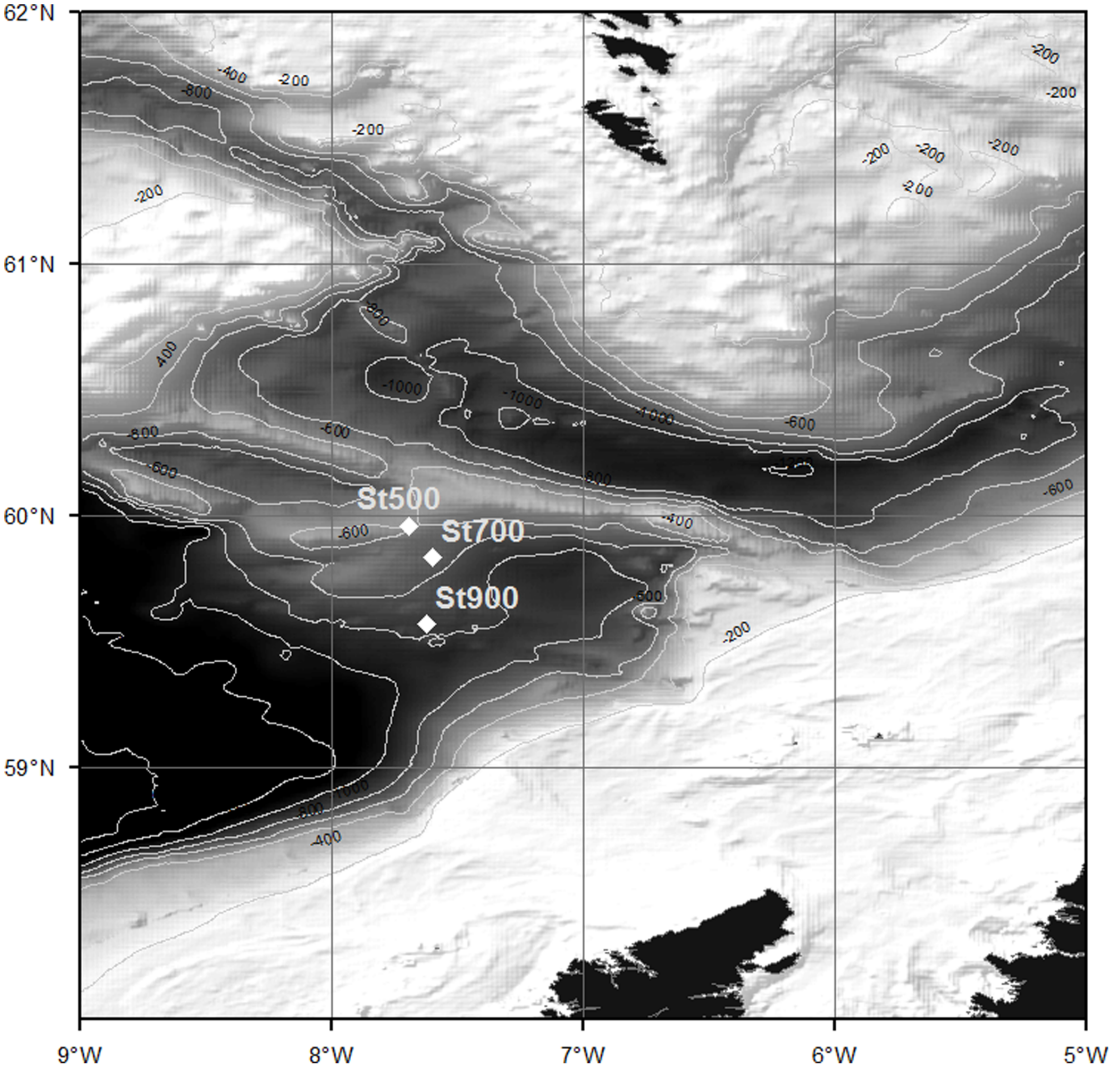
Bathymetric map of study area indicating the sampling stations. Bathymetry data obtained from GEBCO [57].

The study was conducted on the RRS James Cook (cruise 60) in May-June 2011. Sediment cores were retrieved from 3 stations in the northern Rockall Trough, NE Atlantic using the NOC megacorer (core i.d. 10 cm) (Fig.1; Table 1). No specific permissions were required for these locations/activities. The sampling stations were close to but outside the boundaries of the Darwin Mounds Special Area of Conservation and the field study did not involve endangered or protected species. All cores were characterised by the presence of ∼10 cm of sandy sediment overlying glacial mud [28]. Undisturbed sediment cores were transported into a temperature controlled room, set at the in situ temperature of 8°C, immediately after retrieval. Large animals and gravel were removed prior to sediment sectioning. The top 3 cm from 8 sediment cores per station were pooled and homogenised for use in the experiment (see below). Three replicate samples from the homogenised sediment (∼20 ml) were frozen for biogeochemical analysis of bulk sediment (Table 1).

**Table 1:** Station list and sediment characteristics at each station.

Stations	Depth (m)	Lat	Long	TC (%)	TOC (%)	TN (%)	δ^13^C-OM (‰)	δ^13^C-carbonate (‰)
St500	558	59° 57′ 36″ N	7° 41′ 46″ W	3.16	0.15	0.02	−23.6	0.07
St700	741	59° 49′ 55″ N	7° 35′ 56″ W	2.43	0.15	0.02	−23.5	0.25
St900	945	59° 34′ 12″ N	7° 37′ 19″ W	4.11	0.27	0.04	−22.7	−0.01

TC: total carbon, TOC: total organic carbon, TN: total nitrogen, δ^13^C-OM: stable carbon isotope signature of background sediment organic matter, δ^13^C-carbonate: stable carbon isotope signature of background sediment inorganic matter.

### Experimental Set-up

Sediment slurries were prepared in 125 ml amber glass vials (EP Scientific Products) by adding 20 ml of sterile-filtered (0.2 µm) seawater to 20 ml sediment. As a priming agent, we used the ^13^C-labelled diatom *Thalassiosira rotula*, which was cultured and harvested as in Gontikaki et al. [23]. The final ^13^C enrichment of the diatoms was ∼42%. At each station, the incubation vials were divided in 4 treatments (3 replicates per treatment); controls (no diatom addition) were used to determine background mineralisation, and low (LC), medium (MC) and high (HC) treatments contained increasing amounts of diatom detritus. The amount of added diatom C was equivalent to 0.03–0.07 mg C ml^−1^_ws_, 0.12–0.28 mg C ml^−1^_ws_ and 0.31–0.69 mg C ml^−1^_ws_, in the LC, MC and HC treatments respectively. These amounts represent 5% (LC), 20% (MC) and 50% (HC) of the annual C flux at 400 m, 600 m and 800 m in the study area and were calculated based on the depth-dependent export flux equation given in Schlüter et al. [29] and primary productivity values for the NE Atlantic from Sathyendranath et al. [30] (250 g C m^−2^ y^−1^). The simulated deposition of phytodetritus in our experiment exceeded the natural deposition occurring within the 3-week experimental time but covered the whole range of algal C pulses used in previous ITEs at continental slope and abyssal sediments [23], [24], [25], [26], [31]. Due to sampling difficulties, our final sampling stations were deeper than originally planned and although the depth difference between them was still ∼ 200 m, the non-linear decrease in C flux with depth meant that the pre-weighed amount of diatoms per station deviated slightly from the planned annual flux %. The effect was more pronounced in treatment HC which represented 74% of the annual C flux at St500 and 61% at St900 (for comparison, the added C ranged between 6–7.5% and 24–30% of the annual C flux for the LC and MC treatments respectively).

Following the addition of the priming agent, the vials were sealed with screw caps fitted with PTFE/silicone septa and purged for 5 min with a gas mixture of N_2_:O_2_ (80∶20 v:v; BOC). The vials were incubated at 8°C and samples were taken after 7, 14 and 21 days. Due to destructive sampling, a different set of vials were prepared for each sampling time. The vials were manually shaken daily for the duration of the experiment. A set of slurries were prepared and immediately processed to establish initial conditions (start values of concentration and isotopic signature of CO_2_ in seawater and headspace).

### Sample Processing

Sampling for CO_2_ and ^13^CO_2_ analysis in the headspace was performed by removing 12 ml of gas with a gas-tight syringe through the vial septum. The gas sample was transferred to nitrogen-flushed Exetainers® (Labco, U.K) and stored at experimental temperature until analysis. Water samples for dissolved inorganic C (DIC and DI^13^C) analysis were sterile-filtered (0.2 µm) into 3.6 ml Exetainers®, poisoned with 0.2% (vol) mercuric chloride and stored at experimental temperature. DIC samples were quantitatively converted to CO_2_ by acidification before analysis. The concentration and C isotope ratios of CO_2_ either in the headspace samples or in the CO_2_ released by acidification were measured on a Gas-bench II connected to a Delta^Plus^ Advantage isotope ratio mass spectrometer (IRMS; both Thermo Finnigan, Germany). CO_2_ concentrations were calculated from the combined area counts of masses 44, 45 and 46 given in the standard output of the IRMS in the manner of Joos et al. [32] and a calibration curve derived from known DIC concentration standards. Carbon isotopes were calculated using ISODAT NT software version 2.0 (ThermoElectron) and are expressed in the δ notation relative to Vienna Pee Dee Belemnite (VPDB): δ^13^C (‰) = [(R_sample/_R_standard_) –1] x 10^3^, where R_sample_ and R_standard_ are the ^13^C/^12^C of the sample and standard, respectively.

The amount of CO_2_ from priming was determined according to van Nugteren et al. [21]. First, the δ^13^C of total CO_2_ (δ^13^C_total_) was calculated as the concentration-weighed average of that measured in the headspace and water after 1‰ correction for fractionation between CO_2_ gas in the aqueous and gaseous phase [33] as:
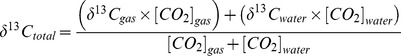
(1)


Subsequently, the δ^13^C of the produced CO_2_ (δ^13^C_prod_) was calculated from the δ^13^C_total_ at the start and end of the incubation as:
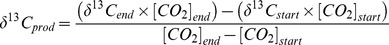
(2)


Finally, a two-end member linear mixing model was used to estimate the maximum amount of CO_2_ originating from carbonate dissolution as:
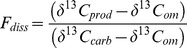
(3)where F_diss_ = [CO_2_]_carb/_[CO_2_]_prod_ and δ^13^C_prod_, δ^13^C_om_ and δ^13^C_carb_ are the carbon isotopic signatures of produced CO_2_, sediment OM and bulk carbonate respectively. The fraction of CO_2_ originating from carbonate dissolution (F_diss_) was subtracted from the CO_2_ produced in control incubations to obtain the background OM mineralisation (OM_miner_control_). The presence of the ^13^C tracer in the LC, MC and HC treatments does not allow the direct calculation of F_diss_. For that reason, the amount of CO_2_ attributed to carbonate dissolution in ^13^C-amended vials was determined from the F_diss_ in the corresponding controls and was proportional to the total CO_2_ production in the vial [21]. To verify the assumption that the proportion of carbonate dissolution will not change because of OM input, we calculated F_diss_ in parallel incubations amended with unlabelled diatoms (St700– MC treatment only). We found no significant difference in the fraction of produced CO_2_ from dissolution caused by diatom addition (65±8% in controls vs. 63±15% in unlabelled diatom-amended treatments).

The total CO_2_ (^13^C+^12^C) originating from the mineralisation of diatom detritus ([CO_2_]_diatom_) was calculated as the product of excess ^13^C and [CO_2_]_end_, divided by the fractional abundance of ^13^C in the algae (0.42). The mineralisation of existing sediment OM in ^13^C-amended treatments was calculated as:
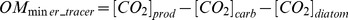
(4)


Finally, priming C-CO_2_ (µg C ml^−1^_ws_) was calculated as the difference in sediment OM mineralisation between ^13^C diatom-amended treatments and controls:
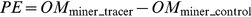
(5)


### Statistical Analysis

The effect of time (continuous variable), treatment (categorical variable, levels: LC, MC, HC) and station (categorical variable, levels: St500, St700, St900) on total and diatom OM mineralisation, and priming intensity was tested using linear regression. A model validation was applied to check that the underlying statistical assumptions were not violated: homogeneity of variance was evaluated by plotting the residuals vs. the fitted values, normality was assessed by plotting the theoretical quantiles vs. the standardised residuals (qq plots) and independence was evaluated by plotting the residuals vs. each explanatory variable. Influential observations were identified using Cook’s distance [34]. Where model validation indicated instances of unequal variance, we used linear regression with the generalised least squares estimation procedure (GLS) [35]. This technique allows the residual spread to vary between individual levels of a particular explanatory variable or combinations of variables. To find the minimal adequate model, we followed the protocol described in Zuur et al. [35]. Initially, the optimal random structure was determined based on the Akaike Information Criteria (AIC) by comparing a model without any variance-covariates (equivalent to linear regression) with subsequent GLS models that contained different variance structures [36]. The selection of the optimal random structure was performed using restricted maximum likelihood estimation. Subsequently, the optimal fixed structure was determined by backward selection using the likelihood ratio (L. ratio) test obtained by the maximum likelihood estimation. The final model, in terms of random and fixed structures was re-fitted using the restricted maximum likelihood estimation to obtain the numerical output and was evaluated as described above. The analysis was performed using the “nlme” package [37] in the “R” programming environment [38].

## Results

### Total Mineralisation

Background mineralisation rate (controls) was significantly higher at St500 compared to deeper stations but did not differ between St700 and St900 (F = 28.55, df_2,24_, p<0.001) (Table 2). The addition of diatoms induced a stepwise increase in total CO_2_ production in MC and HC treatments compared to background levels but low substrate quantity in LC treatment was not sufficient to induce a measurable increase in total CO_2_ production (L = 64.99, df_1_, p<0.001; File S1: Table 1). Total mineralisation rate did not change significantly between days 7 and 21 (L = 0.01, df_1_, p = 0.918). Highest activity was measured in St500 but no difference in total mineralisation was measured between the two deeper stations (L = 45.36, df_1_, p<0.001).

**Table 2:** Average total CO_2_ production and diatom-derived C mineralisation at the end of the experiment.

Station	Treatment	[CO_2_]_prod_ (µg C-CO_2_ ml^−1^_ws_)	[CO_2_]_diatom_ (µg C-CO_2_ ml^−1^_ws_)	[CO_2_]_diatom_ (% of [CO_2_]_prod_)	[CO_2_]_diatom_ (% ofadded C)	Carbonate dissolution* (% of [CO_2_]_prod_)
St500	Control	13.76				52–64
	LC	9.3	3.5	37.5	5.0	
	MC	13.6	5.4	40.0	2.0	
	HC	24.9	7.3	29.2	1.0	
St700	Control	4.2				63–70
	LC	4.9	1.5	30.2	3.5	
	MC	19.0	6.6	34.9	3.9	
	HC	36.2	10.8	30.0	2.5	
St900	Control	7.32				50–66
	LC	7.5	1.4	18.5	4.5	
	MC	12.4	4.6	37.3	3.8	
	HC	25.0	8.9	35.7	2.9	

* calculated from control vials sacrificed at all sampling intervals.

### Mineralisation of Diatom-derived OM

**Figure 2:**
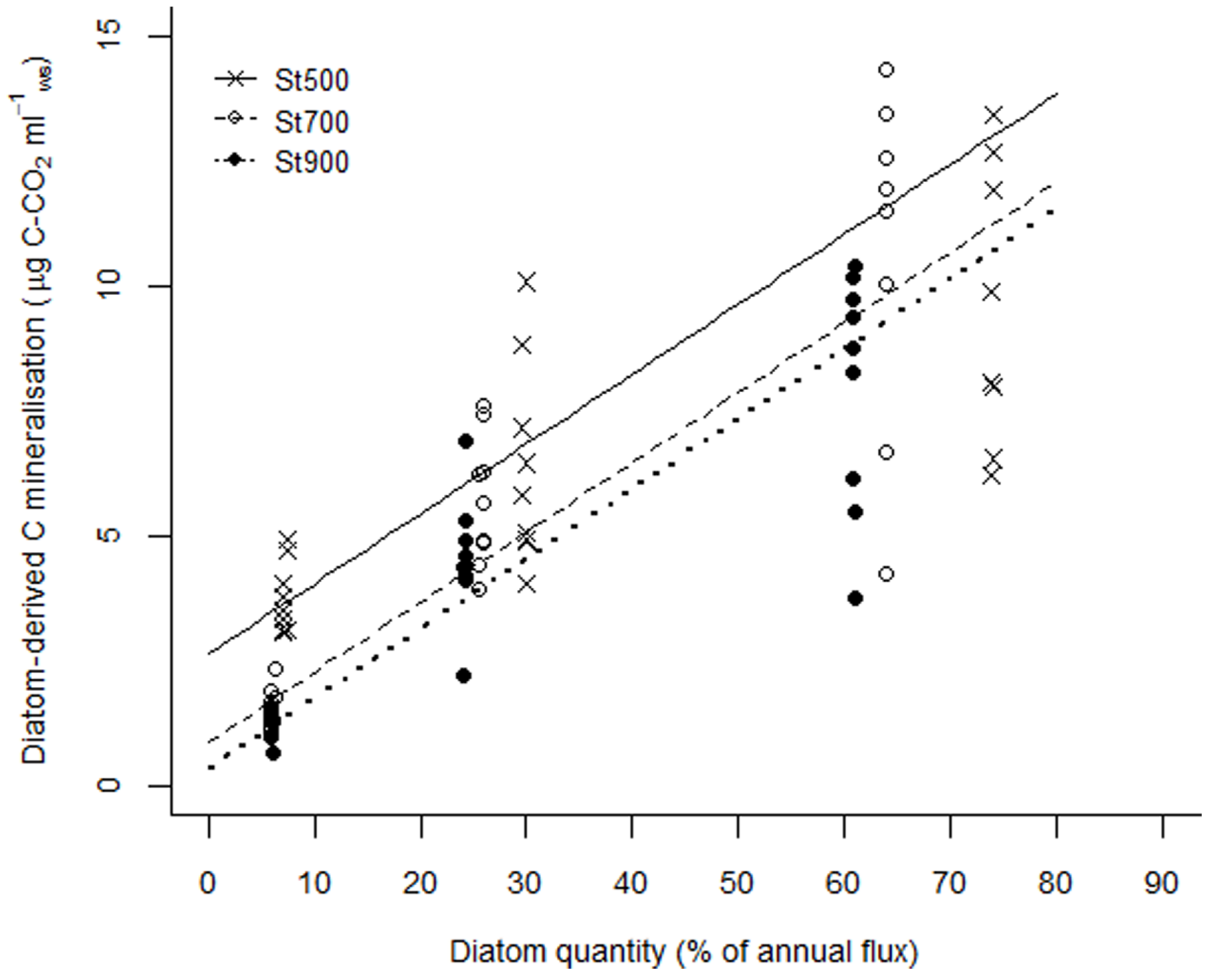
Diatom-derived C mineralisation (µg C-CO_2_ ml^−1^_ws_ ) as a function of substrate quantity. The diatom substrate quantity is expressed as % of annual C flux at the corresponding station.

Mineralisation of diatom OM decreased with depth (L = 55.36, df_2_, p<0.001) and increased with quantity (L = 128.86, df_2_, p<0.001) but did not change significantly between days 7 and 21 (L = 0.28, df_1_ = 1, p = 0.594; File S1: Table 2). In order to standardize for the differences in the absolute amount of added substrate, the same analysis was performed using substrate quantity as % of annual C flux at each station (continuous variable) instead of treatment levels. This model yielded the same outcome, i.e. diatom OM mineralisation increased with substrate quantity as % of annual C flux (L = 92.22, df_1_, p<0.001) and decreased with depth (L = 48.78, df_1_, p<0.001) (Fig.2). After 21 days, mineralisation of diatom C ranged from 30–40% of the total CO_2_ production across treatments and stations with the exception of low treatment at St900 (Table 2). The efficiency of the benthic community to mineralise diatom OM dropped with increasing substrate quantity and [CO_2_]_diatom_ represented <5% of the added C in all cases (Table 2).

### Priming Effect

**Figure 3:**
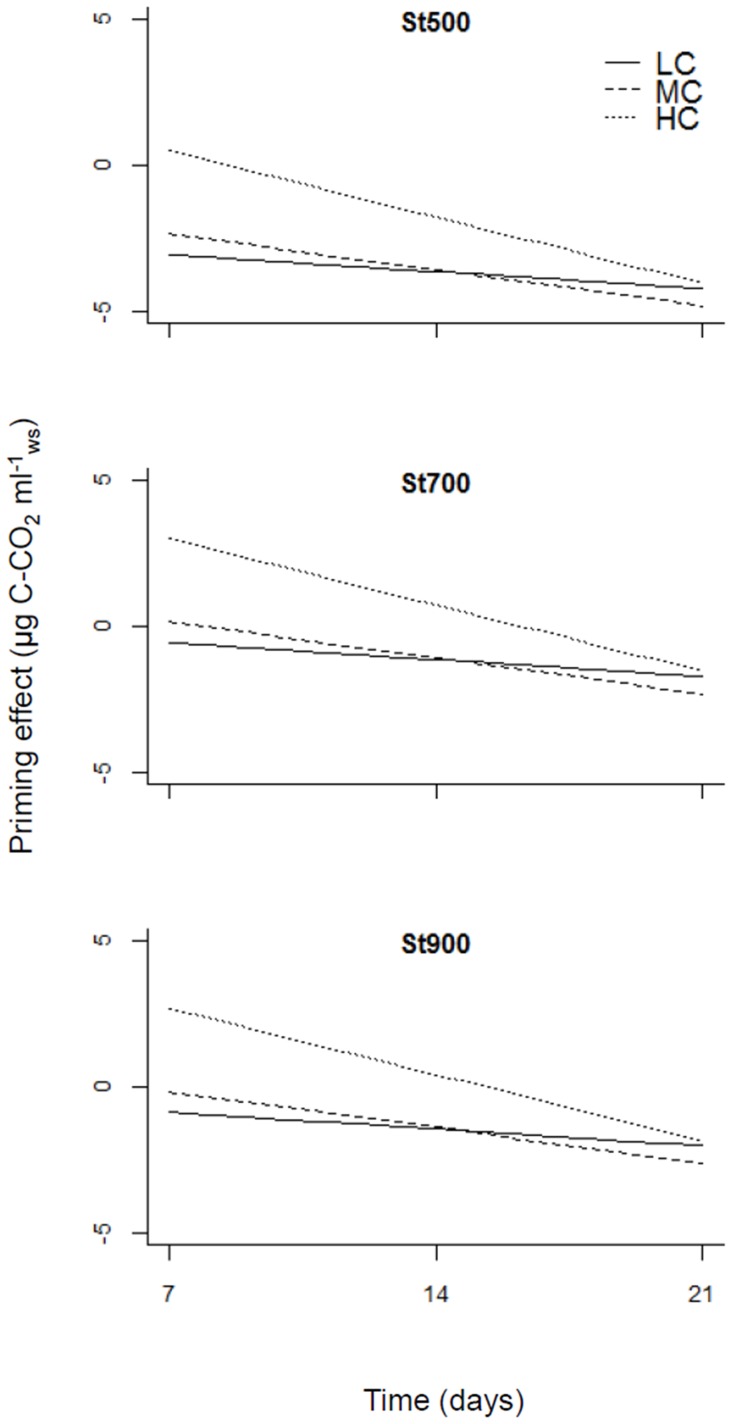
Temporal trends in priming effect (µg C-CO_2_ ml^−1^_ws_) with increasing levels of substrate quantity. LC, MC and HC stand for low, medium and high diatom quantity treatment respectively.

The addition of diatom detritus yielded negative PE on sediment OM mineralisation which was dependant on treatment (Time x Treatment interaction; L = 8.58, df_2_, p = 0.013) and differed with station (L = 34.63, df_2_, p<0.001; File S1: Table 3). The rate of negative priming was significantly different only between LC and HC treatments (t = −2.74, p = 0.008) but this was attributed to differences in PE intensity during the initial stages of the experiment; at the end of the incubation PE was comparable between treatments (Fig. 3). The intensity of negative priming was highest at St500 but did not differ significantly between St700 and St900 (St500–St700: t = 6.81, p<0.001; St500–St900: t = 5.99, p<0.001; St700–St900: t = −0.83, p = 0.405). At the end of the incubation, the decrease in sediment OM mineralisation due to PE corresponded to 60–85% (Table 3).

**Table 3:** Percentage decrease in sediment organic matter mineralisation due to priming effects at the end of incubation time in LC, MC and HC treatments (based on model-predicted values).

Station	% reduction of background OM mineralisation due to PE		
	LC	MC	HC
St500	63	58	65
St700	85	80	77
St900	65	86	84

LC, MC and HC correspond to low, medium and high diatom quantity treatments respectively.

## Discussion

Numerous ITEs have demonstrated the rapid response of deep-sea benthic communities to the arrival of labile OM and the regulation of its intensity by the quantity and quality of the added substrate [23], [24], [25], [26], [31], [39]. The technical challenges of conducting in situ and onboard incubations of undisturbed benthic communities have restricted deep-sea ITEs to short-term incubation times (<7 days, with the exception of Witte et al. [25]) and limited replication and experimental treatments. The slurry incubation methodology is an alternative that allows the study of the combined effects of several factors within a single experimental set-up. Recently, van Nugteren et al. [21] combined slurry incubations with ITEs to study priming effects in intertidal and subtidal coastal sediments. Here, we used the same methodology to generate the most comprehensive dataset so far on the effect of quantity on labile OM mineralisation and occurrence of PEs in continental slope sediments at intermediate time scales. As every experimental approach, slurry incubations involve disadvantages, one of them being their sensitivity to carbonate dissolution due to the possible exhaustion of alkalinity or buffer capacity [21]. However, appropriate corrections for dissolution have been applied and the parallel incubations with unlabelled diatoms confirmed the assumption that carbonate dissolution dynamics did not change with substrate addition. The small sediment amounts used in slurries are not representative of the whole benthic community and mineralisation rates cannot be compared to those obtained by in situ experimentation or whole-core incubations. However, the observed trends in PEs are not invalidated by our methodological approach since this is comparison-based and all data are obtained under the same experimental conditions.

### Mineralisation of Labelled Diatoms

The mineralisation of diatom OM increased with quantity and was higher at St500 although it is not certain whether this is due to higher metabolic rates at shallower depth or the higher relative amount of substrate applied at this station. Previous studies using ^13^C-labelled diatoms to study benthic C cycling in deep-sea [26] and estuarine [40] sediments have also demonstrated a stepwise effect of substrate availability on its mineralisation. The addition of medium and high amounts of diatom C in our study led to a flush of CO_2_ during the first week of incubation possibly corresponding to the “awakening” of dormant or resting microbes into activity [41], [42] and/or a switch in substrate utilization and rapid growth of opportunistic microorganisms capable of utilising the offered substrate [12]. Substrate addition at low levels did not cause a significant increase in total mineralisation suggesting no major changes to ecosystem functioning and microbial community structure in the LC treatment.

**Figure 4:**
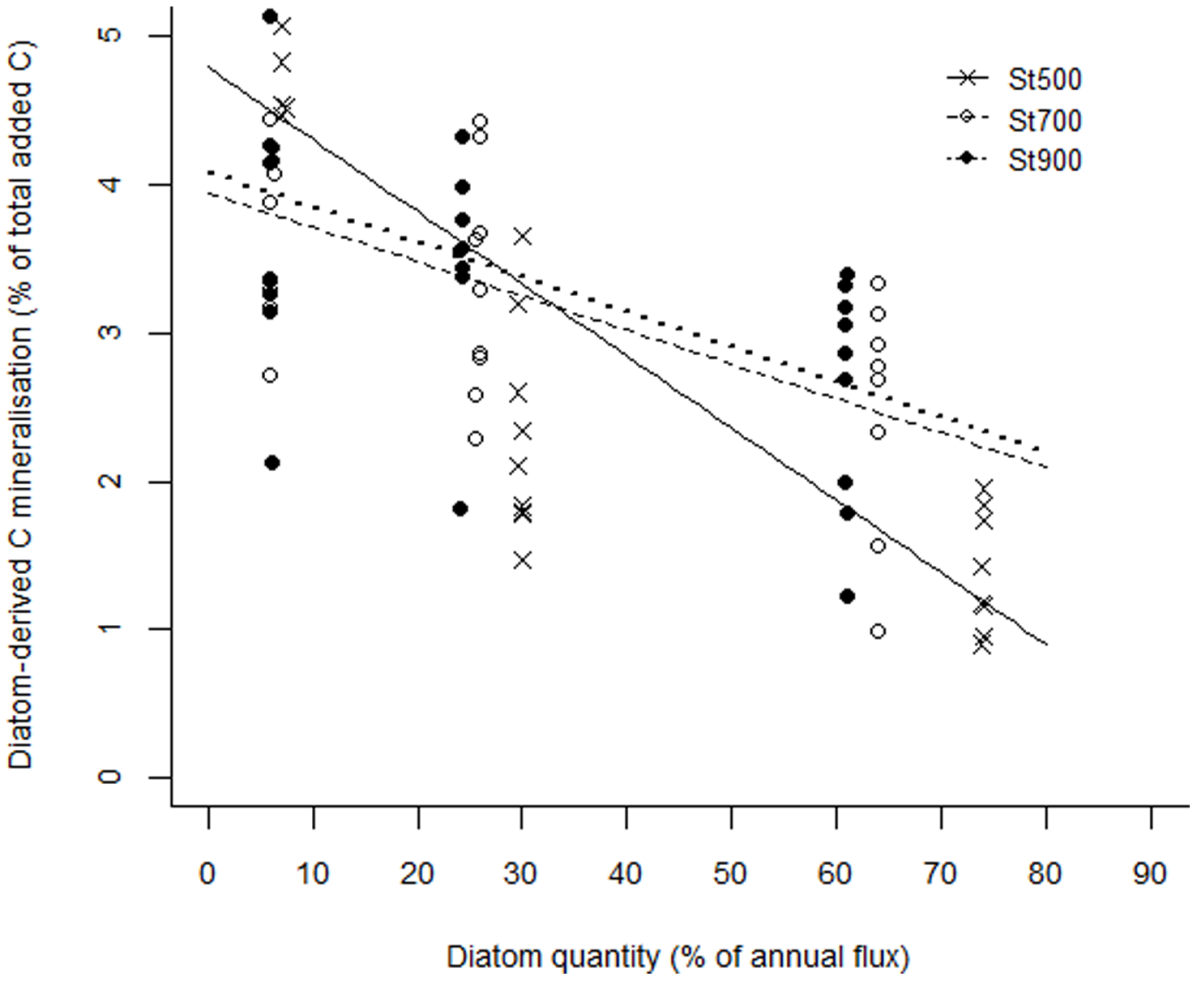
Reduction in diatom-derived C mineralisation efficiency with increasing substrate quantity. Mineralised diatom C is expressed as the percentage of total diatom C added.

The efficiency of the microbial community to mineralise diatom-derived OM decreased with increasing substrate quantity (Fig.4). Similar effects have been observed in estuarine sediments at increasing levels of ^13^C-enriched diatoms addition [40] and soils after addition of increasing amounts of glucose [42], [43]. This could be related to a greater proportion of assimilated C being allocated to maintenance metabolism (respiration) under limited resource availability as opposed to growth [40], [44], [45]. The amount of respired diatom C ranged from 3–5, 2–4 and 1–3% of the total substrate input in LC, MC and HC treatments respectively with the largest fraction mineralised within 7 days (Fig.4). In subtidal and intertidal sediments in the North Sea, van Nugteren et al. [21] and Hansen and Blackburn [46] found that 19–33% and 16–23% of the added phytodetritus was mineralised after 20–21 days respectively. Similarly to our results, 60% [21] and 80–90% [46] of algae mineralisation took place within 7 and 5 days respectively. The above suggest the existence of a particularly labile fraction in phytodetritus which is rapidly mineralised whereas the majority of algal material remains unutilised after several days of incubation. The initial rapid mineralisation of algal substrates could be explained by the addition of low molecular weight dissolved OM, together with the particulate diatom detritus, which is directly assimilated by bacteria without the need of extracellular enzymes, while the mineralisation of the remainder proceeds at a much slower pace [23]. Furthermore, microbial reworking of labile substrates has been shown to produce recalcitrant dissolved OM making it resistant to further degradation [47], [48] and the incorporation of labile C into bacterial structural components contributes to the refractory OM pool in sediments [8], [9]. The change in substrate lability due to microbial processing and secondary production could thus explain the low mineralisation efficiency of algal detritus following the initial rapid mineralisation rates.

### Priming Effects

The addition of labelled diatoms induced negative PE in all treatments, i.e. the mineralisation of the existing sediment OM decreased as a result of enrichment with labile material. Based on model-derived priming C-CO_2_ values, we calculated that the reduction in sediment OC mineralisation after 3 weeks could be up to 86% (Table 3). However, the magnitude of negative PE could be lower since the estimated carbonate dissolution represents a maximum value [21]. At 7 days, PE had a positive value in HC treatments. The positive PE at the initial stages of the incubation was most likely caused by the accelerated turnover or pool substitution of microbial biomass shortly after labile OM input, which was proportional to substrate quantity (“apparent” PE). However, the decrease in sediment OM mineralisation rate was steeper in HC treatment and reached similar levels of negative PE intensity to LC and MC treatments at the end of the experiment suggesting that “real” negative PE was not affected by substrate quantity (Fig.3).

Negative PE in our study is probably attributed to the microbial community switching to the more labile diatom detritus decomposition, a phenomenon called “preferential substrate utilization” (PSU) in soil sciences [49]. PSU and the associated negative PE are often encountered in cases of excessive substrate additions (>200% of microbial biomass) [17], [49], [50]. High diatom quantity resulting in the sudden increase in CO_2_ production in MC and HC could explain the occurrence of PSU due to high substrate addition in these treatments. However this explanation is insufficient to explain PSU in the LC treatment where the quantity of diatoms ranged between 45–70% of microbial biomass [23], [51] and did not cause major changes to the natural functioning of the system, as evidenced by the lack of increase in CO_2_ production following substrate addition. Based on the observations from all treatments, PSU and consequently negative PE in our study are most likely attributed to the particularly low sediment organic C (OC) content at all stations (0.2%) and thus lack of abundant material subject to priming. This hypothesis can also explain the lack of diatom quantity effect on the intensity of negative PE. There is indeed evidence to suggest that OC content plays a role in PE regulation. PEs seem to be more pronounced in C-rich soils, increasing almost linearly with OC content (at least for the investigated range of 2.3–5.1%OC) [52]. This seems to be true even for extremely stable soils; Fontaine et al. [16] observed positive priming of ancient OM (∼2500 year-old C, 3.2%OC) after addition of fresh plant-derived material. On the contrary, the addition of a labile substrate to a particularly C-poor stabilised soil (0.5%OC) led to negative priming [53].

The accumulation of C in sediments is directly related to surface primary productivity, lateral particle transport and sediment texture, with grain size negatively correlated to OC concentration [54], [55], [56]. Continental slopes are considered to be sites of high C deposition as a result of intrinsically high rates of production in the marginal ocean and sediment transport from the shelf, and typically contain 0.5–2%OC [54]. Owing to its geological history and oceanographic regime, our study area is characterised by coarse sediments of particularly low sediment OC content despite moderately high surface productivity in overlying waters [28]. The negative PE observed here could thus be an exception among continental slopes owing to the unusual sediment characteristics of the study area. We postulate that PEs will normally be positive in continental slope sediments and that their intensity will be a direct function of sediment OC content. More experiments with varying supply of substrate targeting C-poor vs. C-rich sediments are needed to confirm these hypotheses.

## Supporting Information

File S1 **Optimal models from the analysis of total mineralisation (** **Table 1** **), diatom OM mineralisation (** **Table 2** **) and priming effect (** **Table 3** **) data for the effect of time (continuous variable), station (categorical variable, levels: St500, St700, St900) and treatment (categorical variable, levels: LC, MC, HC).**
(DOCX)
