# Supplementary material for: Negative Priming Effect on Organic Matter Mineralisation in NE Atlantic Slope Sediments
Source: PLoS One. 2013 Jun 28;8(6):e67722. doi: 10.1371/journal.pone.0067722 (PMC3695930; doi:10.1371/journal.pone.0067722)
Supplement: File S1 — Optimal models from the analysis of total mineralisation ( Table 1 ), diatom OM mineralisation ( Table 2 ) and priming effect ( Table 3 ) data for the effect of time (continuous variable), station (categorical variable, levels: St500, St700, St900) and treatment (categorical variable, levels: LC, MC, HC). (DOCX) [file pone.0067722.s001.docx]

Supplementary material

**File S1:** Optimal models from the analysis of total mineralisation (Table 1), diatom OM mineralisation (Table 2) and priming effect (Table 3) data for the effect of time (continuous variable), station (categorical variable, levels: St500, St700, St900) and treatment (categorical variable, levels: LC, MC, HC).

**Table 1 Optimal model from the total mineralisation analysis.**

| The optimal model was a GLS model that allowed the residual spread to vary by treatment (L = 70.82, df_2_, p<0.001). |
| --- |
| *Total mineralisation_ij_ = intercept + station_ij_ + treatment_j_ + ε_ij_* |

*Total mineralisation_ij_* is the total mineralisation of the *i*th observation in treatment *j* and *ε_ij_= N* (0, σ*_j_*^2^).

**Table 2 Optimal model from the mineralisation of diatom-derived OM analysis.**

| The optimal model was a GLS model that allowed the residual spread to vary by treatment (L = 51.23, df_2_, p<0.001). |
| --- |
| *Diatom mineralisation_ij_ = intercept + station_ij_ + treatment_j_ + ε_ij_* |

*Diatom mineralisation_ij_* is the mineralisation of diatom C of the *i*th observation in treatment *j* and *ε_ij_= N* (0, σ*_j_*^2^).

**Table 3 Optimal model from the priming effect analysis.**

| The optimal model was a GLS model that allowed the residual spread to vary by treatment (L = 17.01, df_2_, p<0.001). |
| --- |
| *Priming effect_ij_ = intercept + time_ij_ + station_ij_ + treatment_j_ + time_ij_: treatment_j_ + ε_ij_* |

*Priming effect_ij_* is the priming effect of the *i*th observation in treatment *j* and *ε_ij_= N* (0, σ*_j_*^2^).
